# Shared and distinct patterns of atypical cortical morphometry in children with autism and anxiety

**DOI:** 10.1093/cercor/bhab502

**Published:** 2022-01-21

**Authors:** Shelly Yin, Seok-Jun Hong, Adriana Di Martino, Michael P Milham, Bo-Yong Park, Oualid Benkarim, Richard A I Bethlehem, Boris C Bernhardt, Casey Paquola

**Affiliations:** Multimodal Imaging and Connectome Analysis Laboratory, McConnell Brain Imaging Centre, Montreal Neurological Institute and Hospital, McGill University, Montreal H3A 2B4, Canada; Center for the Developing Brain and Autism Research Centre, Child Mind Institute, New York City, NY 10022, USA; Center for the Developing Brain and Autism Research Centre, Child Mind Institute, New York City, NY 10022, USA; Center for the Developing Brain and Autism Research Centre, Child Mind Institute, New York City, NY 10022, USA; Multimodal Imaging and Connectome Analysis Laboratory, McConnell Brain Imaging Centre, Montreal Neurological Institute and Hospital, McGill University, Montreal H3A 2B4, Canada; Multimodal Imaging and Connectome Analysis Laboratory, McConnell Brain Imaging Centre, Montreal Neurological Institute and Hospital, McGill University, Montreal H3A 2B4, Canada; Autism Research Center, Cambridge University, Cambridge CB2 2AH, UK; Multimodal Imaging and Connectome Analysis Laboratory, McConnell Brain Imaging Centre, Montreal Neurological Institute and Hospital, McGill University, Montreal H3A 2B4, Canada; Multimodal Imaging and Connectome Analysis Laboratory, McConnell Brain Imaging Centre, Montreal Neurological Institute and Hospital, McGill University, Montreal H3A 2B4, Canada; Institute of Neuroscience and Medicine (INM-1), Forschungszentrum Jülich, Jülich 52428, Germany

**Keywords:** autism, anxiety, cortical thickness, magnetic resonance imaging, structural covariance, transdiagnostic

## Abstract

Autism spectrum disorder (ASD) and anxiety disorders (ANX) are common neurodevelopmental conditions with several overlapping symptoms. Notably, many children and adolescents with ASD also have an ANX diagnosis, suggesting shared pathological mechanisms. Here, we leveraged structural imaging and phenotypic data from 112 youth (33 ASD, 37 ANX, 42 typically developing controls) to assess shared and distinct cortical thickness patterns of the disorders. ANX was associated with widespread increases in cortical thickness, while ASD related to a mixed pattern of subtle increases and decreases across the cortical mantle. Despite the qualitative difference in the case–control contrasts, the statistical maps from the ANX-vs-controls and ASD-vs-controls analyses were significantly correlated when correcting for spatial autocorrelation. Dimensional analysis, regressing trait anxiety and social responsiveness against cortical thickness measures, partially recapitulated diagnosis-based findings. Collectively, our findings provide evidence for a common axis of neurodevelopmental disturbances as well as distinct effects of ASD and ANX on cortical thickness.

## Introduction

Autism spectrum disorder (ASD) and anxiety disorders (ANX) are two of the most prevalent neuropsychiatric conditions affecting young people (Krain et al. 2007; Twenge et al. 2010; Xu et al. 2018; Blumberg et al. 2013) and typically persist into adulthood (Kessler et al. 2005; Mandell et al. 2005). Traditionally, both are diagnosed based on clinical history and symptomatology (Mullin and Funderburk 2013), and their study has provided valuable human evidence on different aspects of social and affective processes. ASD has frequently been associated with atypical social cognition (Frith and Happé 1994; Baron-Cohen 1997), while ANX is related to atypical emotional reactivity and regulation (Campbell-Sills and Barlow 2007; Cisler et al. 2010).

Despite this conceptual distinction, high comorbidities of ASD and ANX render the situation more complicated, with 40% of children and adolescents with ASD having a concurrent ANX diagnosis (van Steensel et al. 2011), which may be indicative of common neurodevelopmental perturbations (White et al. 2009; van Steensel et al. 2011). High comorbidity may also be due to challenges in differential diagnosis using current measures, which adds impetus to studying ASD and ANX in a more dimensional manner. A handful of neuroimaging studies have demonstrated qualitatively distinct effects of ANX and ASD on amygdala volume and task-related activations (Herrington, Maddox, Kerns, et al. 2017; Herrington, Maddox, McVey, et al. 2017; Ibrahim et al. 2019). While these studies focused on localized differences, a more flexible approach may be beneficial to illuminate a broader range of shared or unique aspects of neuroanatomy in ASD and ANX. Cortical thickness is an ideal candidate, because it is widely accessible, provides a clear quantification of brain morphology, and reflects cellular and synaptic organization (Huttenlocher 1979; Schuz and Palm 1989; Desrivieres et al. 2015). Starting from a whole-cortex perspective, we may identify similarities in large-scale patterns, then narrow toward unique neuroanatomical features of the disorders within specific functional systems.

Previous studies have reported cortical anomalies in ASD relative to typically developing controls (TDC) (Redcay 2007; Raznahan et al. 2010; Wallace et al. 2010; Scheel et al. 2011; Khundrakpam et al. 2017; Pereira et al. 2018). While also emphasizing considerable heterogeneity across included sites, several recent large-scale studies have, nevertheless, generally converged on increased thickness in frontal and temporal cortical areas in individuals with ASD (Valk et al. 2015; Hong et al. 2017; Bedford et al. 2020). Studies assessing cortical morphology in ANX have also pointed to increased cortical thickness in medial and lateral frontal regions relative to TDC (Strawn et al. 2014; Gold et al. 2017), with, however, a seemingly different spatial topography compared to ASD. It thus remains to be established whether syndromic differences are also reflected in divergent signatures of regional morphology, and whether the locations of unique neuroanatomical features relate to certain functional systems or types of symptoms.

This study investigated shared and distinct structural substrates of ASD and ANX in the cortex, providing an in vivo neuroanatomical complement to previous clinical and pharmacological studies (van Steensel et al. 2011; Vasa and Mazurek 2015). The importance of community-representative cohorts for translation and inclusivity in psychiatric research has been clearly asserted for clinical trials (Geddes 2005; Surman et al. 2010), but is less acknowledged in neuroimaging research. Based on these recommendations, ANX and attention-deficit/hyperactivity disorder (ADHD) comorbidities were nested within the clinical samples, and we used dimensional correlation analysis and categorical case–control comparisons to balance interpretability and external validity (Kraemer et al. 2004; Kapur et al. 2012). Categorical analyses inform upon the common abnormalities within a primary diagnosis, whereas dimensional analyses illustrate the relevance of cortical variations to a specific clinical symptom.

In light of the comorbidity of ANX in ASD, we predicted shared morphological alterations in both primary diagnostic groups compared to controls. We, nevertheless, also hypothesized that cortical thickness differences would be concentrated within functional networks relevant to disorder-specific behaviors, and these would overlap with dimensional associations of core symptoms. Specifically, we expected more marked structural alterations in ANX within networks previously implicated in emotion processing, such as the limbic and ventral attention networks (Seeley et al. 2007; Menon 2015), and changes related to ASD within networks that may more generally contribute to sociocognitive processing, such as the default mode network (Schilbach et al. 2008). Our work leveraged data provided by the Healthy Brain Network (HBN), an ongoing and large-scale transdiagnostic sample aggregating imaging and phenotypic data in typically developing children and adolescents as well as individuals with a neuropsychiatric diagnosis, that allowed direct comparison across the ASD, ANX, and TDC cohorts (Alexander et al. 2017).

## Materials and Methods

### Participants

We studied the open-access Child Mind Institute HBN dataset (Alexander et al. 2017), which aims to cover a broad range of developmental psychopathology. Participants were recruited via community-referral (for inclusion criteria, see http://fcon_1000.projects.nitrc.org/indi/cmi_healthy_brain_network/inclusion.html). HBN was approved by the Chesapeake Institutional Review Board. Written informed consent was obtained from all participants and from legal guardians of participants younger than 18 years.

The HBN protocol consists of four 3-h sessions collecting general information, behavioral measures, diagnostic assessments, and neuroimaging data (for a complete list of measures, see http://fcon_1000.projects.nitrc.org/indi/cmi_healthy_brain_network/assessments.html). Psychiatric diagnoses were assessed and reported by clinicians according to DSM-5 criteria. Among the 2778 individuals from releases 1–8 with magnetic resonance imaging (MRI), we restricted inclusion to participants with a *T*_1_-weighted image and no diagnosis, ASD diagnosis or ANX diagnosis. Participants were categorized as ASD, if they had any ASD diagnosis, or ANX, if they had any ANX diagnosis without an ASD diagnosis. Of note, individuals in ASD group could also have an ANX diagnosis. Exclusion criteria were any other psychiatric or intellectual comorbidities, except for ADHD in the ASD and ANX groups. We chose to include participants with a secondary diagnosis of ADHD in these groups due to the high prevalence and to provide a more representative community sample of the diagnoses. Although three collection sites provided data to the included releases of the HBN dataset, we further restricted inclusion to participants from the Staten Island (SI) and Rutgers University Brain Imaging Centre (RU) sites, as an adequate number of TDC did not pass quality control in the third site. Following rigorous quality control (see MRI Processing and Quality Control), we included 112 participants: 33 ASD, 37 ANX, and 42 TDC (Table 1).

**Table 1:** Demographic and phenotypic information of participants with a primary diagnosis of ASD or ANX, or TDCs

	Site 1: Staten Island					Site 2: Rutgers University				
	ASD *n* = 18	ANX *n* = 21	TDC *n* = 25	Group difference	Significant post-hoc tests	ASD *n* = 15	ANX *n* = 16	TDC *n* = 17	Group difference	Significant post-hoc tests
Age (years)	11.96 ± 3.53	11.81 ± 2.92	12.50 ± 3.52	*F*(63) = 0.28, *P* = 0.76		12.67 ± 4.61	12.89 ± 3.99	10.48 ± 3.36	*F*(47) = 1.84, *P* = 0.17	
Sex (female)	6	6	13	χ^2^ = 2.97, *P* = 0.26		3	5	9	χ^2^ = 3.96, *P* = 0.13	
ADHD diagnosis	14	11	0	χ^2^ = 28.92, *P* < 0.001	TDC < ASD TDC < ANX	7	14	0	χ^2^ = 25.72, *P* < 0.001	TDC < ASD TDC < ANX ASD < ANX
ANX diagnosis	5	21	0	χ^2^ = 49.03, p < 0.001	TDC < ASD TDC < ANX ASD < ANX	3	16	0	χ^2^ = 37.96, p < 0.001	TDC < ASD TDC < ANX ASD < ANX
WISC	99.91 ± 18.78 (*n* = 9)	98.17 ± 14.84 (n = 12)	106.78 ± 11.75 (*n* = 11)	*F*(31) = 0.84, *P* = 0.44		100.92 ± 14.44 (*n* = 12)	92.79 ± 12.00 (*n* = 14)	94.94 ± 29.83 (*n* = 16)	*F*(41) = 0.50, *P* = 0.61	
SCARED score	23.12 ± 15.01 (*n* = 17)	26.71 ± 9.49 (*n* = 19)	13.13 ± 7.53 (*n* = 20)	*F*(55) = 8.12, *P* < 0.001	TDC < ASD TDC < ANX	21.14 ± 12.24 (*n* = 14)	20.34 ± 9.82 (*n* = 16)	13.38 ± 6.97 (*n* = 16)	*F*(45) = 2.96, *P* = 0.06	
SRS-2 score	94.18 ± 27.18 (*n* = 17)	61.76 ± 24.87 (*n* = 21)	29.96 ± 24.31 (*n* = 23)	*F*(60) = 31.65, *P* < 0.001	TDC < ASD TDC < ANX ANX < ASD	82.92 ± 25.86 (*n* = 12)	52.47 ± 16.97 (*n* = 15)	34.19 ± 23.77 (*n* = 16)	*F*(42) = 16.46, *P* < 0.001	TDC < ASD ANX < ASD

Note: *n* is provided where data were not complete for all participants*.*

### MRI Acquisition

Imaging at SI was conducted using a 1.5 T Siemens Avanto scanner with a 32-channel head coil. Three-dimensional *T*_1_-weighted sagittal magnetization-prepared rapid acquisition gradient echo (MPRAGE) structural images were obtained with the following parameters: repetition time (TR) = 2730 ms, echo time (TE) = 1.64, 3.5, 5.36, or 7.22 ms, flip angle = 7°, field-of-view (FoV) = 256 mm^2^, resulting in 176 slices with 1.0 × 1.0 × 1.0 mm^3^ voxels (Alexander et al. 2017). Imaging at the RUBIC was conducted using a 3 T Siemens Tim Trio scanner with a 32-channel head coil. Three-dimensional *T*_1_-weighted sagittal MPRAGE structural images were obtained with the following parameters: TR = 2500 ms, TE = 3.15 ms, flip angle = 8°, FoV = 256 mm^2^, resulting in 224 slices with 0.8 × 0.8 × 0.8 mm^3^ voxels (Alexander et al. 2017).

### MRI Processing and Quality Control

FreeSurfer (v6.0; http://surfer.nmr.mgh.harvard.edu) was used to generate cortical surface models and to measure cortical thickness (Fischl and Dale 2000). In brief, FreeSurfer automatically reconstructs geometric models of the inner and outer cortical interfaces using a series of volume- and surface-based processing steps. Extracted surfaces in each individual were registered to fsaverage5, an average spherical representation with 20 484 surface points, by aligning cortical folding patterns. Surface extractions were visually inspected, and segmentation inaccuracies were manually corrected by one rater (S.Y.) blinded to participant diagnoses. We excluded 43% of participants because of head motion or low tissue contrast. Thickness data were smoothed using a surface-based Gaussian kernel with 20 mm full-width-at-half-maximum. This process reduces noise and misalignment between vertices by replacing values in images as a weighted average of itself and its neighboring vertices (Lerch and Evans 2005). Subsequent, surface-based analysis was carried out using SurfStat (https://mica-mni.github.io/surfstat/; Worsley et al. 2009) for Matlab (R2017b, The Mathworks).

### Phenotypic Assessments

We focused on the Social Responsiveness Scale (SRS-2) and the Screen for Child Anxiety Related Disorders (SCARED) to index autism and anxiety risk, respectively. Both scales have moderate-to-high internal consistency, interrater reliability, and test–retest reliability (Bölte et al. 2008; Su et al. 2008; Bruni 2014). SRS-2 measures deficits of social interaction and communication in ASD and consists of 65 items rated on a 3-point scale by parents of participants ages 5–17 years (Constantino et al. 2003). SCARED is a questionnaire consisting of 41 items rated on a 3-point scale that screens for childhood anxiety (Birmaher et al. 1999). Parent- and self-report components of SCARED were moderately correlated (*r* = 0.31, *P* < 0.001). In line with prior studies (Gold et al. 2017; Ivarsson et al. 2018), an average score was used. Group differences (ASD, ANX, TDC) in phenotypes (SRS-2 and SCARED) were assessed within each site using one-way analysis of variance. Pair-wise differences were evaluated post-hoc with a series of Tukey tests.

### Brain–Phenotype Analyses

To assess brain–phenotype associations, we fitted linear models to assess effects of SRS-2 (or SCARED) score on cortical thickness measures

$$ {T}_i={\beta}_0+{\beta}_1\ast \mathrm{Sex}+{\beta}_2\ast \mathrm{Age}+{\beta}_3\ast \mathrm{score}+\varepsilon . $$

We included all participants who passed quality control in this analysis. We tested for significant clusters by correcting for multiple comparisons with random field theory (Worsley et al. 2009). This controlled the chance of reporting a family-wise error (FWE) to *P* < 0.05. As in previous study, a cluster defining threshold of *P* < 0.025 was used (Valk et al. 2017).

To assess shared substrates of both SRS-2 and SCARED on brain structure, we computed product–moment correlations between the *t*-statistic maps of the above contrast. Spatial dependencies are produced in cortical measurements by smoothing and motion artifacts, as well as the spatial constraints of brain organization. Parametric tests on the correspondence of spatial maps falsely assume spatial independence, however, leading to high false-positive rates. Thus, we determined the significance of spatial map correspondence using the spatial spin permutation test method with 10 000 permutations (Alexander-Bloch et al. 2018; Vos de Wael et al. 2020). In brief, this method generates a null distribution by comparing a spatial map to a permutated map created by applying random rotational permutations to a spherical representation of a cortical surface (Alexander-Bloch et al. 2018). We deemed the association significant where *P*_spin_ < 0.025.

### Case–Control Differences in Cortical Thickness

The matchit package in R (v3.2.5; https://cran.r-project.org/web/packages/MatchIt/MatchIt.pdf) was used to match the participants across groups in order to reduce model dependence and potential for bias (Ho et al. 2007). Given that the ASD group had fewest participants, we matched the TDC group to the ASD group based on age and sex and matched the ANX group to the ASD group based on age, sex, and ADHD diagnosis. The procedure was conducted independently within each site. The argument specifications were “nearest” for method with a ratio of 1, indicating that participants should be matched as closely as possible and only once. The matched cohorts used for within site case–control contrasts consisted of 54 participants for site 1 and 45 in site 2. Site 1 and site 2 each had equal representation of ASD, ANX, and TDC groups. Table 2 summarizes changes in the groups before and after matching.

**Table 2:** Balance of age, sex, and comorbidity of ADHD before/after matching

		Site 1: Staten Island			Site 2: Rutgers University		
		Unmatched	Matched	Balance improvement	Unmatched	Matched	Balance improvement
Age (years)	ASD	11.96	11.96		12.67	12.67	
	ANX	11.81	12.30	−127%	12.88	12.75	66%
	TDC	12.50	12.69	−34%	10.48	10.93	20%
Sex (female)	ASD	6	6		3	3	
	ANX	6	6	100%	5	4	41%
	TDC	13	6	100%	9	7	19%
ADHD	ASD	14	14		7	7	
	ANX	13	11	35%	14	13	2%

Note: ANX and TDC groups were independently matched to the ASD group (fewest participants), as such the ASD group distributions do not change with the matching procedure.

Linear models compared cortical thickness at each vertex *i* between ANX and TDC as well as ASD and TDC. The corresponding model at each vertex was

$$ {T}_i={\beta}_0+{\beta}_1\ast \mathrm{Sex}+{\beta}_2\ast \mathrm{Age}+{\beta}_3\ast \mathrm{group}+\varepsilon . $$

We enacted this model within each site using matched data. We tested for significant clusters by correcting for multiple comparisons with random field theory (Worsley et al. 2009). This controlled the chance of reporting a FWE to *P* < 0.05. As in previous work, a cluster defining threshold of *P* < 0.025 was used (Valk et al. 2017).

We also repeated the model twice with both sites combined. In one iteration we harmonized across sites using ComBat (Johnson et al. 2007), a powerful technique for batch-effect correction that estimates site-specific scaling factors and uses empirical Bayes to improve the estimation for small sample sizes (Fortin et al. 2018), and in another iteration we simply regressed site within the linear model

$$ {T}_i={\beta}_0+{\beta}_1\ast \mathrm{Sex}+{\beta}_2\ast \mathrm{Age}+{\beta}_3\ast \mathrm{group}+{\beta}_4\ast \mathrm{site}+\varepsilon . $$

To determine the spatial correspondence of *t*-statistic maps, we performed pair-wise product–moment correlations between case–control *t*-statistic maps (ASD-TDC and ANX-TDC), as well as dimensional *t*-statistic maps (SRS-2 and SCARED), and evaluated significance with spin permutation testing, as in Brain-Phenotype Analyses.

### Common and Distinct Patterns in Functional Networks

The canonical seven functional networks (Yeo et al. 2011) provide a framework to localize shared and distinct patterns of ASD and ANX to specific brain systems. First, we defined types (cross-disorder, categorical vs. dimensional, or between-site) for the key spatial correspondence tests. The type names reflect the parameter that differs between the two maps. For example, comparison of site 1 ASD-TDC with site 1 ANX-TDC is a cross-disorder test, because only the disorder contrast is different between the maps, while the approach and the site are the same for both maps. Four tests belong to each type. Next, we repeated each of the typified tests within each functional network. In other words, we performed pair-wise product–moment correlations between *t*-statistics within each functional network. Then, we calculated the average and standard deviation of correlation coefficients for each type and each network to determine whether spatial correspondence was specific to certain functional systems.

To examine distinct patterns of the disorders, we performed linear regression between pairs of *t*-statistic maps (ASD-TDC vs. ANX-TDC; SRS-2 vs. SCARED) and extracted the standardized residuals to represent the deviation of a region from the common axis of the two maps. The procedure was performed in each site separately, then residuals were averaged across the two sites. The average residual maps were thresholded based on consistency across sites. Specifically, vertices with residuals of different signs in each site were set to zero, thereby maintaining residuals in regions with a consistent direction of the effect. To test for preferential localization of distinct effects within certain functional networks, we compared the average residual within each network to 10 000 spin permutations of the residual map. We deemed the association significant where *P*_spin_ < 0.025.

## Results

### Group Differences in Phenotypes

**Figure 1:**
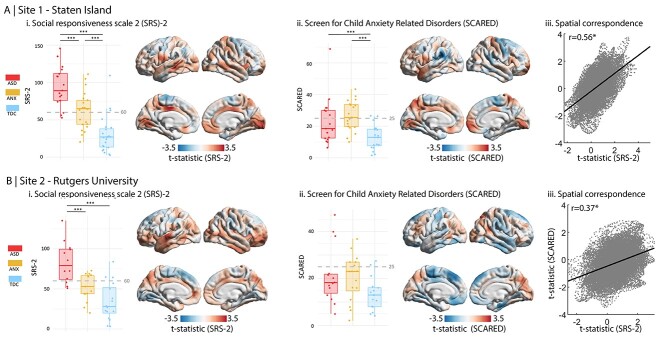
Dimensional analyses of ASD- and ANX-related risk. Boxplots show the range of (i) SRS-2 and (ii) SCARED scores within each group, ^*^^*^^*^P < 0.001. Cutoff scores for risk of ASD and ANX are shown on the boxplots (60 for SRS-2 and 25 for SCARED; Birmaher et al. 1997, Constantino et al. 2007; Canals et al. 2012; Moody et al. 2017). Surface plots show the main effect of (i) SRS-2 and (ii) SCARED scores on cortical thickness. (iii) Scatter plots show spatial map correspondence between the main effect of SRS-2 and the main effect of SCARED.

Studying phenotypic differences among ASD, ANX, and TDC in site 1 (i.e., Staten Island), we observed group differences in SRS-2 (*F*(2, 60) = 31.65, *P* < 0.001) and SCARED (*F*(2, 55) = 8.12, *P* < 0.001) (Fig. 1*A***;**  Table 1). Post-hoc Tukey tests showed a graded increase in SRS-2 from TDC to ANX to ASD. ASD and ANX did not differ on SCARED, but both were significantly greater than TDC. Repeating the analysis without the outlier did not impact the reported effects. Analyzing site 2 (i.e., Rutgers) yielded similar results. Group differences were found in SRS-2 (*F*(2, 42) = 16.46, *P* < 0.001) and post-hoc Tukey tests showed that scores in ASD were significantly greater than both ANX and TDC groups (Fig. 1*B***;**  Table 1). Analysis of variance did not indicate significant differences between groups for SCARED (*F*(45) = 2.96, *P* = 0.06), but both ASD and ANX groups were greater than TDC at a trend level.

### Associations between Phenotypic and Cortical Thickness Measures

At the neuroanatomical level, we correlated SRS-2 and SCARED scores with cortical thickness across all three diagnosis-based groups (Fig. 1*A*). Higher scores were variably associated with increased and decreased cortical thickness but no clusters passed the criteria for significance after correcting for multiple comparisons. The SRS-2 and SCARED *t*-statistic maps were moderately correlated (*r* = 0.56) in site 1 (Fig. 1*A*iii), suggesting convergence of the SRS-2 and SCARED associations with cortical thickness. Spin permutation testing confirmed that overlap was not attributable to shared spatial autocorrelation (*P*_spin_ = 0.002). We also observed significant correspondence between SRS-2 and SCARED *t*-statistic maps in site 2 (*r* = 0.37, *P*_spin_ = 0.006) (Fig. 1*B*iii).

### Group Differences in Cortical Thickness

**Figure 2:**
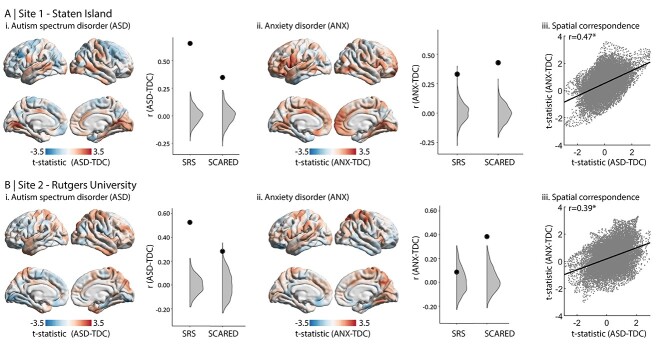
Case–control analysis. Cortical thickness comparison of TDC with (i) ASD and (ii) ANX. The neighboring plots show the correlation coefficient of the correspondence between each group difference map with each phenotypic map (from Fig. 1). The large black dots indicate the empirical correlation coefficient, while the gray areas represent the null distribution. Thereby, when the black dot is higher than the gray area, the correlation is significant. (iii) Scatter plots show spatial map correspondence between ASD-vs-TDC and ANX-vs-TDC.

In both sites, case–control differences were associated with varied increases and decreases in cortical thickness (Fig. 2). No clusters passed threshold for significance after correcting for multiple comparisons. Combining the two datasets, no clusters passed the threshold for significance, either when harmonizing the data across sites or regressing site in the linear model. Despite evident site-wise differences, we consistently observed correspondence of the ASD-vs-TDC and ANX-vs-TDC *t*-statistic maps (site 1: *r* = 0.47. site 2: *r* = 0.39), supporting our hypothesis of common axis of neurodevelopmental abnormalities in ASD and ANX. Spin permutation tests indicated that this similarity was not attributable to shared spatial autocorrelation (Fig. 2iii; *P*_spin_ < 0.001).

Furthermore, in site 1, the ASD-vs-TDC contrast exhibited moderate–strong correspondence with the main effect of SRS-2 on cortical thickness (Fig. 2*A*, *r* = 0.65; *P*_spin_ < 0.001), suggesting convergent cortical substrates of phenotypic variables of autism risk and autism diagnosis. Specificity for ASD was suggested, as the SRS-2 correlation map was only weakly correlated with the ANX-vs-TDC contrast map (Fig. 2*A***;**  *r* = 0.33; *P*_spin_ < 0.001). Considering the main effect of SCARED on cortical thickness, the *t*-statistic map was moderately correlated with the ANX-vs-TDC contrast (*r* = 0.42; *P*_spin_ < 0.001) and weakly correlated with the ASD-vs-TDC contrast (*r* = 0.34, *P*_spin_ < 0.001). Overall similar, albeit weaker, findings were seen in site 2 (Fig. 2*B*). The *t*-statistic maps of SRS-2 and ASD-vs-TDC effect were moderately correlated (*r* = 0.53; *P*_spin_ < 0.001), while no correspondence with ANX-vs-TDC was indicated (*r* = 0.09 *P*_spin_ = 0.133). Moreover, the *t*-statistic map of the SCARED effect was moderate–weakly correlated with both the ANX-vs-TDC contrast (*r* = 0.38, *P*_spin_ < 0.001) and the ASD-vs-TDC comparison (*r* = 0.28, *P*_spin_ = 0.006).

### Combining Brain–Phenotype and Case–Control Analyses

**Figure 3:**
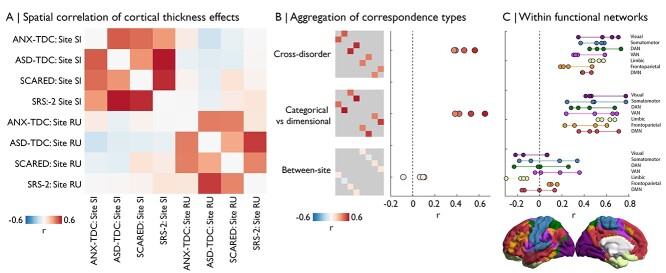
Comparison and aggregation of spatial maps. (*A*) The matrix depicts the spatial correlation of case–control and dimensional maps across sites. (*B*) Labeling the cross-correlation estimates by analysis type, we can observe consistency of cross-disorder similarities and categorical-vs-dimensional approaches, whereas similarities across sites are consistently low. (*C*) Cross-correlations performed within functional network (Yeo et al. 2011), organized by type. DAN = dorsal attention network. VAN = ventral attention network. DMN = default mode network.

We sought to aggregate analyses to determine the consistency of our key results. Cross-correlation of the eight maps shown in Figures 1–2 highlights common effects, despite site-related idiosyncrasies (Fig. 3*A*). We observed moderate spatial correlations of ASD-related and ANX-related cortical thickness differences, regardless of site or whether a categorical or dimensional approach was used (*r* = 0.45 ± 0.08; Fig. 3*B*). Additionally, we found moderate spatial correlations between cortical thickness differences of categorical and dimensional approaches, regardless of site or whether the predictor focused on ASD or ANX (*r* = 0.50 ± 0.12; Fig. 3*B*). Furthermore, to test whether correspondences were specific to certain functional networks, we repeated analyses using cross-correlation of *t*-statistics within functional networks (Yeo et al. 2011). We observed overlapping effect sizes across all networks, showing distribution of the correspondences across the brain (Fig. 3*C*).

### Distinct Patterns within Functional Networks

**Figure 4:**
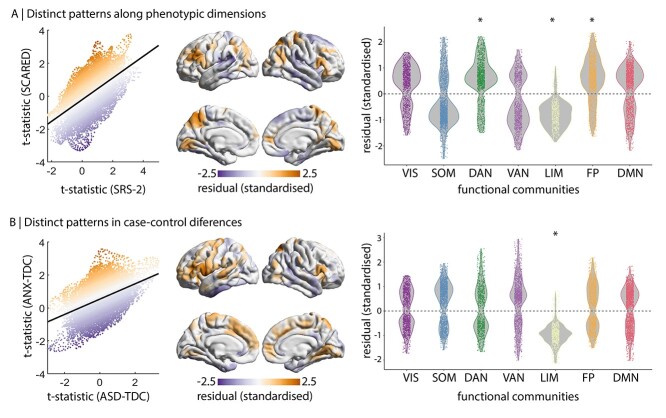
Distinct cortical thickness signatures within functional networks. *Left* correspondence of *t*-statistic maps for site 1, colored by standardized residuals. *Centre* averaged, consistency-thresholded standardized residuals, taken across both sites. *Right* residual stratified by functional networks (Yeo et al. 2011). ^*^P_spin_ < 0.05. VIS = visual. SOM = somatomotor. DAN = dorsal attention network. VAN = ventral attention network. LIM = limbic. FP = frontoparietal. DMN = default mode network. (*A*) Comparison of SRS-2 and SCARED maps. (*B*) Comparison of ASD-TDC and ANX-TDC maps.

The preceding analyses highlighted moderate spatial correspondence of ASD and ANX associations with cortical thickness, using both dimensional and case–control approaches. Certain regions exhibit distinctively stronger effects in one phenotype or one disease, which is reflected by the residuals of linear models (Fig. 4). To robustly define distinctive regions, we thresholded the residual map based on consistency across the two sites. Stratifying residuals by functional networks, dorsal attention and frontoparietal cortical thickness showed stronger increases related to SCARED than SRS-2 (dorsal attention: residuals = 0.55 ± 0.81, *P*_spin_ = 0.035; frontoparietal: residuals = 0.56 ± 0.83, *P*_spin_ = 0.013; Fig. 4*A*). Conversely, the limbic network showed stronger effects related to SRS-2 than SCARED (residual = −0.78 ± 0.51, *P*_spin_ = 0.049; Fig. 4*A*), which reflects the positive association of SRS-2 and negative association of SCARED with cortical thickness in the limbic network (Fig. 1). Similarly, limbic network showed stronger effects related to ASD than ANX (residual = −0.95 ± 0.46, *P*_spin_ = 0.013; Fig. 4*B*), reflecting a slightly positive association of ASD and slightly negative association of ANX with cortical thickness in the limbic network (Fig. 2). Thus, neuroanatomy of the limbic network may dissociate ASD and ANX.

## Discussion

Our study aimed at identifying shared and distinct cortical substrates of primary diagnoses of ASD and ANX as well as phenotypic risk measures. Case–control differences in cortical thickness significantly overlapped, demonstrating a common axis of ANX- and ASD-related cortical malformations, though cortical thickness signatures in the limbic network differed between the disorders. Our case–control findings were complemented by phenotypic correlation analyses, lending a dimensional perspective on structural substrates of ASD and ANX risk. Behavioral phenotypes captured similar morphological patterns as group-level differences, particularly in the case of ASD. In addition, we identified a unique signature of the anxiety phenotype in increased cortical thickness within dorsal attention and frontoparietal networks. Collectively, our neuroanatomical findings support emerging transdiagnostic frameworks in highlighting common substrates of neurodevelopmental disorders.

Our regional analysis harnessed MRI-based cortical thickness measures, a reliable technique that has previously been applied to profile morphological variations across a broad spectrum of typical and atypical neurodevelopment (Raznahan et al. 2010). In line with prior surface- and voxel-based analyses, our results were suggestive of diffuse gray matter increases in individuals with ASD (Raznahan et al. 2010; Wallace et al. 2010; Scheel et al. 2011; Valk et al. 2015; Hong et al. 2017; Khundrakpam et al. 2017; Pereira et al. 2018; Bedford et al. 2020), as well as ANX (Strawn et al. 2014; Gold et al. 2017). However, sensitivity of the present transdiagnostic sample was relatively low for specifying localized differences. While limitations in sensitivity may result from our modest sample size, our study benefitted from strict inclusion criteria with respect to data quality, together with formal matching procedures that ensured similar age and sex distributions across the cohorts as well as a matched prevalence of ADHD comorbidity in ASD and ANX groups. Furthermore, increasing the sample size, by combining the sites and performing batch-effect harmonization (Johnson et al. 2007; Fortin et al. 2018), did not provide significant clusters of case–control differences. Heterogeneity in cortical thickness estimates within each group were pronounced. Further stratification of diagnostic groups may facilitate identification of regional disruptions, as has been shown in ASD (Hrdlicka et al. 2005; Hong et al. 2019; Chen et al. 2019). As the HBN cohort increases, normative modeling approaches could be used to approximate deviations within the TDC across age, then individual deviations from normative curves may be estimated for ASD and ANX (Marquand et al. 2016; Bethlehem et al. 2020).

In addition to the categorical case–control analyses, we examined the association of regional markers of cortical thickness with behavioral risk indices of ASD and ANX. Diagnostic and phenotypic associations closely overlapped in the case of ASD but were less convergent in the case of ANX. This suggests that SRS-2 captures a relationship between cortical morphology and clinical phenotype that transcends diagnostic boundaries but is most severe in ASD. SCARED scores were not significantly correlated with cortical thickness across the cohort. Two previous studies with larger sample sizes of typically developing children (Merz et al. 2018) or both typically developing children and children with an ANX (Gold et al. 2017) also reported a null relationship of SCARED with vertex-wise estimates of cortical thickness, suggesting the lack of brain–behavior relationship is not simply due to sample size or the use of a transdiagnostic cohort. As this approach aggregates multiple forms of anxiety, more fine-grained phenotyping may be necessary to identify brain–behavior associations of anxiety. This may be especially crucial in transdiagnostic research programs, where diagnostic categories are associated with distinct forms of anxiety.

The diagnostic groups share a common axis of cortical thickness differences relative to TDC. This was confirmed by spin tests that control for the shared spatial autocorrelations in two surface-based maps (Alexander-Bloch et al. 2018) and was observed within both sites. Genome-wide association studies indicate that major neuropsychiatric disorders have overlapping polygenic risk profiles (Cross-Disorder Group 2013). These genetic polymorphisms can perturb neurodevelopment in a regionally specific manner, providing a plausible mechanism for the emergence of common axis of cortical abnormalities across ASD and ANX. The overlap observed in the present study may also be driven by a subgroup within the ASD cohort with high ANX. Several studies have demonstrated neuroanatomical subtypes of ASD (Hrdlicka et al. 2005; Hong et al. 2019; Chen et al. 2019), although the relation to ANX remains unclear as it is often an exclusion criterion. Alternatively, the common axis may reflect shared symptoms that are not associated with ASD or ANX specifically, such as attention deficits and hyperactivity. While each of these possibilities is concordant with the hypothesis that genetic similarities underlie the common axis of cortical abnormalities, it remains to be seen whether this spans the two diagnoses and is related to specific subtypes or shared symptoms.

While ASD and ANX share a common axis of morphological abnormalities, we observed their dissociation in the limbic network. Using both case–control and dimensional approaches, ASD (or ASD-related risk) was associated with slight increases in thickness, whereas ANX (or ANX-related risk) was associated with slight decreases in thickness. Localizing this divergence was enabled by focusing on effects that were consistent across both sites and by expanding our field of view from vertices to functional networks. Notably, the limbic network, encompassing the temporal pole and orbitofrontal cortex, is strongly connected to the amygdala (Kerestes et al. 2017). Previous studies on the dissociation of ASD and ANX centered on the amygdala, and indeed suggested disorder-related differences (Herrington, Maddox, Kerns, et al. 2017; Herrington, Maddox, McVey, et al. 2017; Ibrahim et al. 2019). Thus, our findings add to a growing body of evidence on limbic system dissociation between ASD and ANX.

As the HBN spans many neurodevelopmental disorders, there are only modest sample sizes of individuals diagnosed with specific disorders who met our inclusion criteria. Given these limitations, we have focused on effects that were replicable in both sites, such as the common axis of case–control differences in ASD and ANX. Shared ADHD comorbidities may partially account this overlap, however, we lacked the sample size to test this hypothesis with sensitivity analyses. Nevertheless, including individuals with multiple diagnoses is an important step toward more inclusive research that is generalizable to clinical populations. The proportion of individuals with ASD and ADHD in the present study resides within population estimates [37% (Gadow et al. 2006) and 85% (Lee and Ousley 2006)]. Our dimensional analysis further aimed to address high rates of comorbidity and substantial within-group heterogeneity. Additional questionnaires should be analyzed to determine whether questionnaires targeted at different behavioral dimensions of ASD and ANX capture other brain–behavior associations and disentangle anxious symptoms in ASD from ANX.

Our assessment of regional morphology lends support to the power of transdiagnostic approaches to unveil neurobiological factors that may play a role in the risk for prevalent mental health conditions such as ASD and ANX. The present study demonstrates that primary diagnoses of ASD and ANX relate to distinctive patterns of cortical morphology, even though many individuals shared comorbid disorders. Further understanding the neuroanatomical intersections and divergences of overlapping neurodevelopmental disorders will be benefitted by the continued growth of open access transdiagnostic datasets.

## Funding

Autism Research Trust and British Academy Fellowship (PF2\180017 to R.A.I.B.); National Institute of Mental Heath (NIMH-105506 and NIMH-099059, respectively, to A.D. and M.M.); Fonds de la Recherche du Québec—Santé (FRQ-S) to C.P.; Canadian Institutes of Health Research (FDN-154298), SickKids Foundation (NI17-039), Natural Sciences and Engineering Research Council (NSERC; Discovery-1304413), Azrieli Center for Autism Research of the Montreal Neurological Institute (ACAR), FRQ-S (Chercheur Boursier Junior 1) to B.C.B.; MNI-Cambridge collaboration grant to R.A.I.B. and B.C.B.

## Notes

The authors would like to thank the Healthy Brain Network for providing the data for the present study. S.Y. received a scholarship from the Natural Sciences and Engineering Research Council of Canada. *Conflict of Interest*: None declared.
